# An Alteration in the Cecal Microbiota Composition by Feeding of 1-Kestose Results in a Marked Increase in the Cecal Butyrate Content in Rats

**DOI:** 10.1371/journal.pone.0166850

**Published:** 2016-11-18

**Authors:** Takumi Tochio, Yasuyuki Kitaura, Saki Nakamura, Chie Sugawa, Motoki Takahashi, Akihito Endo, Yoshiharu Shimomura

**Affiliations:** 1 B Food Science Co., Ltd., Chita, Aichi, Japan; 2 Laboratory of Nutritional Biochemistry, Department of Applied Molecular Biosciences, Graduate School of Bioagricultural Sciences, Nagoya University, Nagoya, Aichi, Japan; 3 Department of Food and Cosmetic Science, Faculty of Bioindustry, Tokyo University of Agriculture, Abashiri, Hokkaido, Japan; Kobe University, JAPAN

## Abstract

Functional food ingredients, including prebiotics, have been ardently developed for improving the intestinal environment. Fructooligosaccarides (FOS), including fructans, are the well researched and commercialized prebiotics. However, to our knowledge, few studies have been conducted on the physiological effects of each component of FOS as prebiotics. 1-Kestose, a component of FOS, is composed of one glucose and two fructose molecules, and is considered as a key prebiotic component in short-chain FOS. In the present study, we examined the effects of dietary 1-kestose using 0.5–5% 1-kestose diets on cecal microbiota composition and cecal contents of short-chain fatty acids and lactate in rats. The findings indicate that dietary 1-kestose induced cecal hypertrophy and alterations in the cecal microbiota composition, including a marked increase in the cell number of Bifidobacterium spp. These alterations were associated with significant increases in acetate and lactate, and a marked increase in butyrate in cecal contents. Furthermore, dietary 1-kestose induced a significant decrease in serum insulin concentration in rats fed 2.5–5% 1-kestose diet. These findings suggest a potential of 1-kestose to be a prebiotic for improving the metabolism of the host.

## Introduction

Recent studies have revealed that human intestinal microbiota has a large impact on the health of the host, and that irregularity of microbiota is linked with lifestyle-related and immunological diseases, such as diabetes, adiposeness, allergy and inflammatory disorders [1,2]. Several environmental factors, including aging, antibiotic usage, and diet, are known to influence the composition of microbiota. Of these factors, diet appears to be the most promising factor for the regulation of health conditions.

A prebiotic is now defined by FAO as “a non-viable food component that confers a health benefit on the host associated with modulation of the microbiota” [3]. Oligosaccharides are usually used as prebiotics and are important food ingredients. Fructooligosaccharides (FOS) are the well commercialized and researched prebiotics and several beneficial properties have been reported. Daily intake of FOS increases levels of fecal bifidobacteria which is associated with a trend toward a relative increase in specific immune response [4], delays the onset of senescence including learning and memory disorders in senescence-accelerated mice [5], prevents the incidence of aberrant crypt foci in mice [6], and modulates cytokine secretion in human peripheral blood monocyte [7]. FOS consist of different ratios of 1-kestose, nystose, and fructofranosylnystose, which have 1–3 fructose monomers linked with sucrose via β2,1 glycosidic bonds [8,9]. Therefore, the key components of prebiotic activity have not been well characterized in FOS. Our recent in vitro study using several oligosaccharides and lactobacilli suggested that 1-kestose content was crucial for prebiotic activity in FOS [10]. On the other hand, in vivo studies using 1-kestose are quite limited; supplementation with 1-kestose in the diet for pregnant and lactating mice increased the IgA levels in maternal milk [11]. Oligosaccharides are usually metabolized in gut microbiota, resulting in accumulation of short-chain fatty acids (SCFA), which have various beneficial effects on the host [12]. Moreover, certain prebiotics have been shown to have positive somatic effects in the host [13]. The purpose of this study was to elucidate the unique somatic effects of 1-kestose and its responsible mechanisms.

## Materials and Methods

### Animal experiments

All procedures for animal experiments in the present study were approved by the Animal Care Committee of Graduate School of Bioagricultural Sciences, Nagoya University. 1-Kestose (purification > 98%) was provided by B Food Science Co., Ltd. (Aichi, Japan). Five experimental diets (Table 1) were prepared in a pellet form by CLEA Japan (Tokyo, Japan): the composition of the control diet was based on the AIN-93G diet, and sucrose in the diet was replaced with the same amount of 1-kestose to create 1-kestose diets at 0.5%, 1.0%, 2.5%, or 5.0% (Table 1).

**Table 1:** Experimental diets.

Ingredient	Control diet (0% 1-kestose)	1-Kestose diet (% 1-kestose)			
		0.5%	1%	2.5%	5%
(g/100 g diet)					
**Corn starch**	51.9486	51.9486	51.9486	51.9486	51.9486
**α-Corn starch**	1.0	1.0	1.0	1.0	1.0
**Sucrose**	10.0	9.5	9.0	7.5	5.0
**Casein**	20.0	20.0	20.0	20.0	20.0
**Soybean oil**	7.0	7.0	7.0	7.0	7.0
**Cellulose**	5.0	5.0	5.0	5.0	5.0
**Mineral mix**	3.5	3.5	3.5	3.5	3.5
**Vitamin mix**	1.0	1.0	1.0	1.0	1.0
**L-Cystin**	0.3	0.3	0.3	0.3	0.3
**Choline bitartrate**	0.25	0.25	0.25	0.25	0.25
**tert-Butylhydroquinone**	0.0014	0.0014	0.0014	0.0014	0.0014
**1-Kestose**	0	0.5	1.0	2.5	5.0
**Total**	100.0	100.0	100.0	100.0	100.0

Forty male Sprague-Dawley rats aged 8 weeks were obtained from Japan SLC (Hamamatsu, Japan) and were individually housed in wire-mesh cages in a conventional animal room with a controlled temperature (23 ± 1°C) and a 12-h light-dark cycle (lights on at 8:00 am). After acclimatization to the animal room for 1 week, the rats were randomly allocated to five groups (n = 8 per group): control (0%), 0.5%, 1.0%, 2.5%, and 5.0% 1-kestose diet groups. The average body weight in each group was 293–295 (± 3–4 standard error) g. Rats in each group were provided free access to water and the corresponding experimental diets for 4 weeks. Food intake and body weight were recorded once a week. The feces were collected during daytime on the day before sacrifice. On the final day of the experiment, rats were sacrificed under anesthesia with isoflurane and blood samples were obtained from the posterior vena cava with a syringe to prepare serum and post-heparin plasma. Subsequently, ceca and cecal contents were rapidly recovered, frozen in liquid nitrogen, and stored at −80°C until analyses.

### Measurement of SCFA, lactate, and blood components

Measurement of SCFA (acetate, propionate, isobutyrate, butyrate, isovalerate, and valerate) were performed by GC/MS (Shimazu, Kyoto, Japan) on Rtx-1701 coloums (Restec, Bellefonte, USA). Preparation of GC/MS samples was performed as follows: 100 mg (wet weight) of cecal contents were suspended in 500 μl pure water. The suspension was stirred for 3 min, followed by the addition of 20 μL of 35% HCl and 500 μL of diethyl ether. After centrifugation at 5000 g for 3 min at 4°C, an upper layer (diethyl ether) was filtered with a polyvinylidene difluoride membrane which had a 0.45 μm pore size (MilliporeSigma, Darmstadt, Germany), the filtrate was used as samples for GS/MS analysis. Measurement of lactate was performed using an F-kit for lactate (Roche Diagnostics GmbH, Basel, Switzerland.

Measurements of concentrations of serum total cholesterol (TC), triglyceride (TG), insulin, and plasma glucose were conducted by SRL Inc. (Tokyo, Japan).

### Analysis of intestinal microorganisms in rat cecal contents

Analyses of five groups of intestinal microorganisms (Bacteroides spp., Bifidobacterium spp., Lactobacillus spp., Clostridium cluster XIVa, and Akkermansia muciniphila) were conducted by Technosuruga Laboratory Co., Ltd. (Shizuoka, Japan). Details of analyses were as follows: the genomic DNA extraction from rat cecal contents and quantitative real-time PCR (qPCR) were performed according to the methods of Takahashi et al. [14]. Primers for qPCR and cycle conditions are indicated in Table 2. Each 16S rDNA of Bacteroides fragilis DSM 2151^T^, Bifidobacterium longum subsp. longum JCM 1217^T^, Clostridium clostridioforme JCM 1291^T^, Lactobacillus casei JCM 1134^T^, and Akkermansia muciniphila ATCC BAA-835^T^ were used as standard curves. 16S rDNA copy numbers were represented as log_10_.

**Table 2:** Primers and program conditions for real-time PCR.

Target	Primer name	Oligonucleotide sequence	PCR program
Bacteroides genus	HuBac594Bhqf (modified)	GTTGTGAAAGTTTGCGGCTCAACC	95°C (5 sec)– 60°C (30 sec) / 35 cycles
	HuBac692r	CTACACCACGAATTCCGCCT	
Bifidobacterium genus	Bif LM 26F	GATTCTGGCTCAGGATGAACGC	95°C (5 sec)–60°C (20 sec)-72°C (20 sec) / 35 cycles
	Bif 228R	CTGATAGGACGCGACCCCAT	
Clostridium cluster XIVa	CXIV-F1	GAWGAAGTATYTCGGTATGT	95°C (5 sec)-52°C (20 sec)-72°C (20 sec) / 35 cycles
	CXIV-R2	CTACGCWCCCTTTACAC	
Lactobacillus genus	LactoR’F	CACAATGGACGMAAGTCTGATG	95°C (5 sec) - 56°C (20 sec)-72°C (50 sec) / 35 cycles
	LBFR	CGCCACTGGTGTTCTTCCAT	
A. muciniphila	Akk-F	CAGCACGTGAAGGTGGGGAC	95°C (5 sec)-57°C (30 sec)-72°C (60 sec) / 35 cycles
	Akk-R	CCTTGCGGTTGGCTTCAGAT	

### Statistical analyses

Each value represents the mean ± SE. Statistical analyses were performed using the StatView (version 5.0) software (SAS Institute, Cary, NC). The data of body weight and food intake, cecum and cecal contents, SCFA and lactate in cecal contents, and concentrations of blood components were analyzed using one-way ANOVA followed by Dunnett’s test to compare the difference between control group and experimental groups. Comparisons of the numbers of intestinal microorganism were performed using Kruskal-Wallis test followed by Mann-Whitney U-test to compare the difference between control group and experimental groups for Bacteroides spp. and Bifidobacterium spp., and species in the Clostridium cluster XIVa, and using Mann-Whitney U-test only for Lactobacillus spp. and A. muciniphila. P values less than 0.05 were considered significant.

## Results

### Effects of dietary 1-kestose on rat body weight, food intake, and weights of cecum and cecal contents

Supplementation of 1-kestose at 0.5–5% into the diet had no effects on body weight of rats on the final day of the experiment or food intake during the 4-week experimental period (Table 3), indicating that supplementation of 1-kestose up to 5% had no effect on the growth of rats.

**Table 3:** Body weight and food intake.

	Control group	0.5% group	1% group	2.5% group	5% group
**Body weight (g)**	481 ± 9	465 ± 11	489 ± 15	477 ± 11	460 ± 8
**Food intake (g/day)**	25.2 ± 0.5	24.6 ± 0.6	25.8 ± 0.9	25.5 ± 0.9	25.1 ± 0.5

Values represent the means ± SE, n = 8. Food intake is the average of 4 weeks of the experimental period.

**Fig 1:**
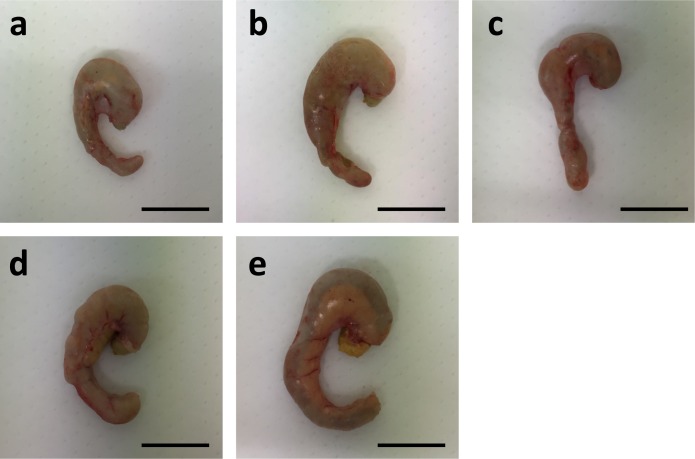
**(a)-(e). Photographs of the cecum with cecal contents in rats.** The cecum from each group of rats: (a) control, (b) 0.5%, (c) 1%, (d) 2.5%, (e) 5%. Bars: 2 cm.

Since 1-kestose is not readily digested in the small intestines of rats and humans [15,16], it is likely metabolized by large intestinal microbiota. In rats, the cecum is the main site for microbial fermentation. The cecum weights of rats in the 1-kestose diet groups gradually increased in a dose-dependent manner (Table 4); supplementation of 1-kestose in the diet, even at 0.5%, significantly increased the cecum weight, and 5% supplementation diet enlarged the cecum ~1.6-fold greater than the control diet. Although the cecal content was significantly increased in the 5% 1-kestose group compared to the control group (Table 4), hypertrophy of the cecum with the contents was apparent even in the 0.5% 1-kestose group and was clear in the 5% 1-kestose group (Fig 1).

**Table 4:** Weights of the cecum and cecal contents.

	Control group	0.5% group	1% group	2.5% group	5% group
Wet weight (g)					
**Cecum**	0.64 ± 0.03	0.74 ± 0.02	0.77 ± 0.03*	0.90 ± 0.03*	1.02 ± 0.04*
**Cecal content**	3.83 ± 0.67	3.71 ± 0.32	3.49 ± 0.24	3.81 ± 0.27	5.73 ± 0.58*

Values represent the means ± SE, n = 8.

* Significant difference compared to the control group (P < 0.05).

The supplementation of 1-kestose to the diet had effects on consistency of the feces in a dose-dependent manner: the color of the feces was black in the control group and beige in the 5% 1-kestose group, and the moisture of the feces collected during daytime on the day before sacrifice was about 7% greater in 5% 1-kestose group than in control group (55 ± 4% vs. 48 ± 3%, respectively).

### The effects of dietary 1-kestose on intestinal microorganisms

The impact of administration of 1-kestose on cecal microbiota was studied by quantitative PCR assay. All of the 1-kestose administration groups (0.5–5% 1-kestose) showed significantly larger numbers of Bacteroides spp. and Bifidobacterium spp., and species in the Clostridium cluster XIVa than those of the control group (Table 5). Specifically, the cell number of Bifidobacterium spp. was more than 7,000-fold greater in the 5% 1-kestose group than in the control group. On the other hand, there was no significant difference in numbers of Lactobacillus spp. or A. muciniphila between control and 5% 1-kestose groups (Table 5).

**Table 5:** Levels of bacterial cell numbers (log_10_ cells/g) in cecal contents.

Bacterial group	Control group	0.5% group	1% group	2.5% group	5% group
**Bacteroides spp.**	8.64	8.98*	9.10*	9.34*	9.05*
	(8.35–8.83)	(8.76–9.30)	(8.91–9.50)	(9.00–9.73)	(8.90–9.28)
**Bifidobacterium spp.**	7.05	8.86*	8.94*	9.95*	10.91*
	(6.99–7.18)	(8.77–8.89)	(8.86–9.09)	(9.09–10.51)	(10.73–10.93)
**Clostridium cluster XIVa**	9.74	11.28*	11.70*	11.74*	10.4*
	(9.52–9.81)	(11.21–11.44)	(11.39–11.80)	(11.73–11.80)	(10.26–10.50)
**Lactobacillus spp.**	8.78	N.D.	N.D.	N.D.	8.89
	(8.55–8.92)				(8.75–9.06)
**A. muciniphila Valerate**	8.93	N.D.	N.D.	N.D.	8.87
	(8.77–9.23)				(8.58–9.24)

Results are represented as the log_10_ of copy number of 16S rDNA per gram of cecal contents.

IQR in parentheses indicates the interquartile range.

*Significant difference compared to control group (P < 0.05).

N.D. represents not determined.

### SCFA and lactate in cecal contents

**Fig 2:**
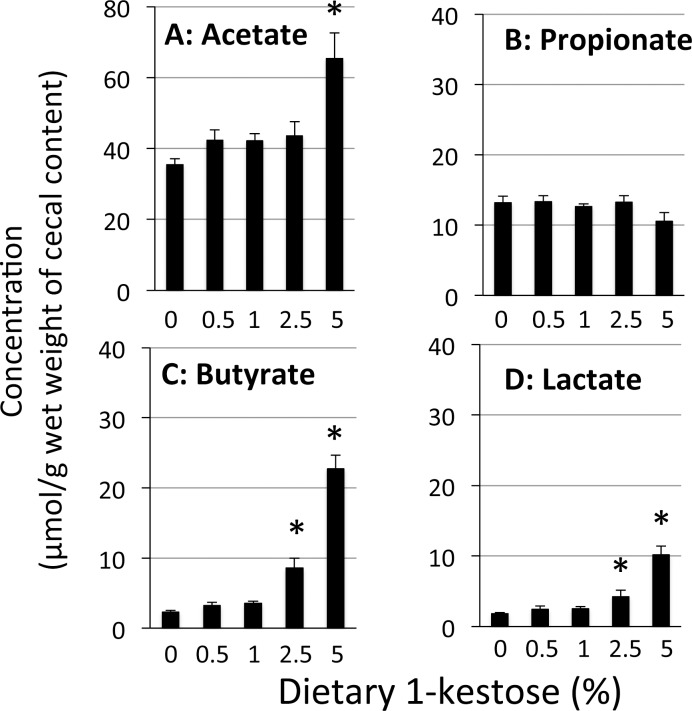
Measurement of SCFAs in cecal contents. Values represent the means ± SE (n = 8), * P <0.05 vs. control group.

Concentrations of SCFA (acetate, propionate, isobutyrate, butyrate, isovalerate, and valerate) and lactate in cecal contents were determined in all of the dietary groups. It is known that the major SCFA in cecal contents are acetate, propionate, and butyrate. Among these SCFA, the concentrations of acetate and butyrate were increased by 1-kestose supplementation in a dose-dependent manner: the acetate concentration was significantly higher in the 5% 1-kestose group than in the control group, and the butyrate concentration was significantly higher in the 2.5% and 5% 1-kestose groups than in the control group (Fig 2). The butyrate concentration level in the 5% 1-kestose group was ~10-fold higher than in the control group. The concentrations of isobutyrate, isovalerate, and valerate in the cecal contents tended to be decreased by supplementation of 1-kestose, although the levels of these fatty acids were relatively low among SCFAs (Table 6).

**Table 6:** Concentrations of isobutyrate, isovalerate, and valerate in cecal contents.

Component	Control group	0.5% group	1% group	2.5% group	5% group
cecal content (μmol/g)					
**Isobutyrate**	0.93 ± 0.06	0.73 ± 0.03*	0.50 ± 0.05*	0.33 ± 0.05*	0.42 ± 0.05*
**Isovalerate**	0.55 ± 0.08	0.36 ± 0.03*	0.24 ± 0.03*	0.20 ± 0.02*	0.35 ± 0.02*
**Valerate**	0.91 ± 0.08	0.78 ± 0.07	0.69 ± 0.08	0.56 ± 0.07*	0.48 ± 0.06*

Values represent the means ± SE, n = 8

* Significant difference compared to control group (P < 0.05).

The lactate concentration in the cecal contents was increased by supplementation of 1-kestose in a dose-dependent manner and was significantly higher in the 2.5% and 5% 1-kestose groups than in the control group (Fig 2).

### Concentrations of blood components

Concentrations of blood TC, TG, glucose, and insulin were measured in all of the dietary groups of rats (Table 7). The TC concentration tended to be decreased by supplementation of 1-kestose, but the decreases were not statistically significant. Blood TG and glucose concentrations were not affected by supplementation of 1-kestose. The insulin concentration was decreased by supplementation of 1-kestose in a dose-dependent manner and was significantly lower in 2.5% and 5% 1-kestose groups than in the control group.

**Table 7:** Concentrations of total cholesterol, triglyceride, glucose, and insulin.

	Control group	0.5% group	1% group	2.5% group	5% group
**Total cholesterol (mg/dL)**	102 ± 4	99 ± 3	94 ± 6	90 ± 6	82 ± 6
**Triglyceride (mg/dL)**	277 ± 17	291 ± 24	270 ± 20	283 ± 20	247 ± 24
**Glucose (mg/dL)**	207 ± 11	202 ± 10	192 ± 11	202 ± 4	188 ± 7
**Insulin (ng/mL)**	4.34 ± 0.51	3.04 ± 0.38	3.08 ± 0.31	2.56 ± 0.31*	2.01 ± 0.24*

Values represent the means ± SE, n = 8.

* Significant difference compared to the control group (P < 0.05).

## Discussion

Recent studies have suggested that intestinal microbiota are closely linked with the development of allergies, chronic gut disorders, and metabolic syndrome [17,18,19]. Since diet has a great impact on the development of well-balanced microbiota, functional food ingredients, including prebiotic oligosaccharides, which can promote the growth of beneficial gut commensals without digestion by the host, are promising for the prevention of disorders. FOS are the well studied and commercialized prebiotics, and has several beneficial effects, such as the promotion of bifidobacteria growth [20]. FOS commonly contain 1-kestose, nystose and fructosyl-nystose at a ratio of 3:6:1 [10]. Each oligosaccharide usually possesses different prebiotic potentials in terms of growth stimulation of beneficial microbes, and it has been reported from in vitro studies that short-chain FOS stimulate the growth of butyrate-producing bacterial strains and Bifidobacterium spp. [21], and that stimulation of growth of intestinal lactic acid bacteria is much greater in 1-kestose than in nystose and fructosyl-nystose [10,22]. In the present study, we demonstrated that feeding of a 1-kestose diet increased weights of the cecum and cecal contents, likely due to the fermentation of 1-kestone in the cecum. 1-Kestose was not detected in cecal contents (data not shown), suggesting that 1-kestose was quickly degraded by microbiota. Our findings show that the growth of Bifidobacterium spp. in cecal contents was greatly enhanced by 1-kestose-supplemented diets, and that their quantity in rats fed a 5% 1-kestose diet was over 7000-fold that of the control group. Bifidobacteria are beneficial gut microbes found in various animals, including humans, and their activity is closely linked with the health of the host [23]. It is known that Bifidobacterium spp. produces acetate and lactate by degradation of oligosaccharides [24]. In fact, acetate and lactate concentrations in cecal contents were significantly increased in rats fed a 5% 1-kestose diet. Furthermore, the 5% 1-kestose diet significantly increased the number of Clostridium cluster XIVa, which produce butyrate via metabolism of sugars and lactate in the gut. Among these combinations of alterations in microbiota composition and their metabolites, butyrate concentrations in cecal contents were significantly elevated by 1-kestose-supplemented diets in rats.

Butyrate is known to have several beneficial effects in the host, including being an energy source for epithelial cells [25], induction of colonic regulatory T cells [26], induction of apoptosis in human colonic carcinoma cells [27], inhibition of inflammatory responses in intestinal biopsy specimens [28], and improvement of metabolic syndrome [29]. Therefore, increased production of butyrate in the intestines is one of the most important beneficial effects of oligosaccharides on human health [30]. Previous studies have also reported increased levels of butyrate production after supplementation of prebiotic oligosaccharides in rats; butyrate production was increased approximately 5.1-fold and 2.4-fold by 10% FOS and 10% galacto-oligosaccharides diets, respectively [31].

Since the metabolizable energy of FOS was a half of that of regular carbohydrates [4], the adverse effect of 1-kestose might be possible in energy intake. The body weight of rats appears to be slightly lower in the 5% 1-kestose group than in the control group. However, the difference in the body weight was not significant, indicating no adverse effect of 1-kestose in the present study.

Among the blood components measured, the concentration of insulin was decreased by supplementation with 1-kestose into the diet in a dose-dependent manner, and the decreases were significant in the 2.5–5% 1-kestose diet groups, suggesting that 1-kestose may raise insulin sensitivity in rats. This effect of 1-kestose might be attributed to the formation of butyrate as previously reported [30]. The concentration of TC tended to be decreased by intake of 1-kestose diet, although the decrease was not statistically significant. Since the excretion of bile acids into the feces is one of the factors to affect the blood concentration of TC, the bile acids in the feces collected on the day before sacrifice of rats in control and 5% 1-kestose diet groups were extracted by the reported method [32] and determined by the direct spectrophotometric method [33]. However, the content of the bile acids in the feces was not different between two groups (10.7 ± 2.5 vs. 10.0 ± 2.7 μmol/g dry feces, respectively). Further studies are required to confirm the effects of 1-kestose on the concentrations of blood components.

Overall, the findings of the present study demonstrated the strong bifidogenic activity of 1-kestose, which was associated with several beneficial effects in the host, increased level of cecal butyrate and decreased level of serum insulin. These findings suggest that 1-kestose may be a promising prebiotic for the treatment of metabolic diseases. It has been reported from the clinical study that 1-kestose exerts a beneficial effect on the clinical symptoms in infants with atopic dermatitis [34]. Further studies including human clinical trials are needed to determine the exact effects of 1-kestose in humans.
